# The complete chloroplast genome sequence of the medicinal plant *Strophanthus divaricatus* (Lour.) Hook. & Arn. (1837) and its phylogenetic analysis

**DOI:** 10.1080/23802359.2026.2638668

**Published:** 2026-03-05

**Authors:** Weiqun Zhou, Juanyu Liu, Xianglan Liang, Yihong Luo, Gouan Shen, Lichai Yuan

**Affiliations:** a Institute of Medicinal Plant Development, Chinese Academy of Medical Sciences & Peking Union Medical College, Beijing, P.R. China; b Hainan Branch of the Institute of Medicinal Plant Development, Chinese Academy of Medical Sciences & Peking Union Medical College, Hainan, P.R. China; c Department of Orthopaedic Surgery, Faculty of Medicine, University of Malaya, Kuala Lumpur, Malaysia; d School of Chemistry and Chemical Engineering, Guangdong Pharmaceutical University, Guangzhou, P.R. China

**Keywords:** Chloroplast, *Strophanthus divaricatus*, plastome, phylogenetic analysis

## Abstract

*Strophanthus divaricatus* (Lour.) Hook. & Arn. (1837) is an important medicinal plant utilized in traditional Chinese and Yao ethnomedicine. Here, we report its complete chloroplast genome based on Illumina sequencing. The circular genome is 155,409 bp in length, with an overall GC content of 38.28%, and displays a typical quadripartite structure comprising a large single-copy region (85,683 bp), a small single-copy region (18,182 bp), and two inverted repeats (25,772 bp each). A total of 130 genes were annotated, including 85 protein-coding genes, 37 tRNAs, and 8 rRNAs. Phylogenetic analysis revealed that *S. divaricatus* is closely related to *S. wallichii*.

## Introduction

*Strophanthus divaricatus* (Lour.) Hook. & Arn. (1837) is a perennial plant belonging to the family Apocynaceae. Southern China—Hainan, Yunnan, Guizhou, Guangdong, and Fujian—and Southeast Asian countries like Laos and Vietnam are home to this species (Li, Leeuwenberg, Middleton 1995). It grows in hilly or mountainous areas, along highways with sparse arboreal cover, or among bushes on inclines. Yao ethnomedicine uses *S. divaricatus*, a traditional Chinese medicine plant. Cardiovascular glycosides, triterpenoids, flavonoids, anthraquinones, alkaloids, and fatty acid chains comprise *S. divaricatus* (Wen 2013). This plant’s roots, stems, leaves, flowers, and seeds are used therapeutically. It has cardiotonic, bradycardia, diuretic, sedative, anti-edema, antibacterial, antioxidant, and anticancer properties (Haux 1999). Traditional medicine treats snakebites, dermatophytic infections, scabies, bone fractures, poliomyelitis sequelae, and many abscesses with *S. divaricatus* (Medicine NUoC 2005). It treats chronic heart failure, arrhythmias, atherosclerosis, severe injuries, and rheumatoid arthritis (Li, Leeuwenberg, and Middleton 1995).

The chloroplast genome (cpDNA) is a crucial component of plant photosynthesis. It is characterized by a relatively low mutation rate, maternal inheritance, and a highly conserved structure (De Las Rivas, Lozano, and Ortiz 2002). The chloroplast genome consists of both coding and non-coding regions. Its sequence variants provide high-resolution molecular evidence for taxonomic classification and genetic differentiation among closely related species (Dobrogojski, Adamiec, Luciński 2020). In recent years, significant advancements in chloroplast genomics have been achieved, greatly enhancing our understanding of plant adaptability, phylogeny, and evolution (Daniell et al. 2016).

Although *S. divaricatus* demonstrates notable pharmacological properties and substantial medicinal potential, its genetic foundation has not been extensively studied, particularly due to the lack of available chloroplast genome data. This knowledge gap obstructs thorough phylogenetic analysis and comparative genomic investigations. Consequently, our research seeks to address this gap by sequencing and examining the entire chloroplast genome of *S. divaricatus*. Moreover, the chloroplast genome data will aid in the conservation and sustainable use of this significant plant species.

This study concentrates on the sequencing, assembly, and annotation of the whole chloroplast genome of *S. divaricatus* to augment its genetic database. A phylogenetic analysis of the family Apocynaceae was conducted to investigate the interrelationships among species.

## Materials and methods

### Plant material collection

**Figure 1.**
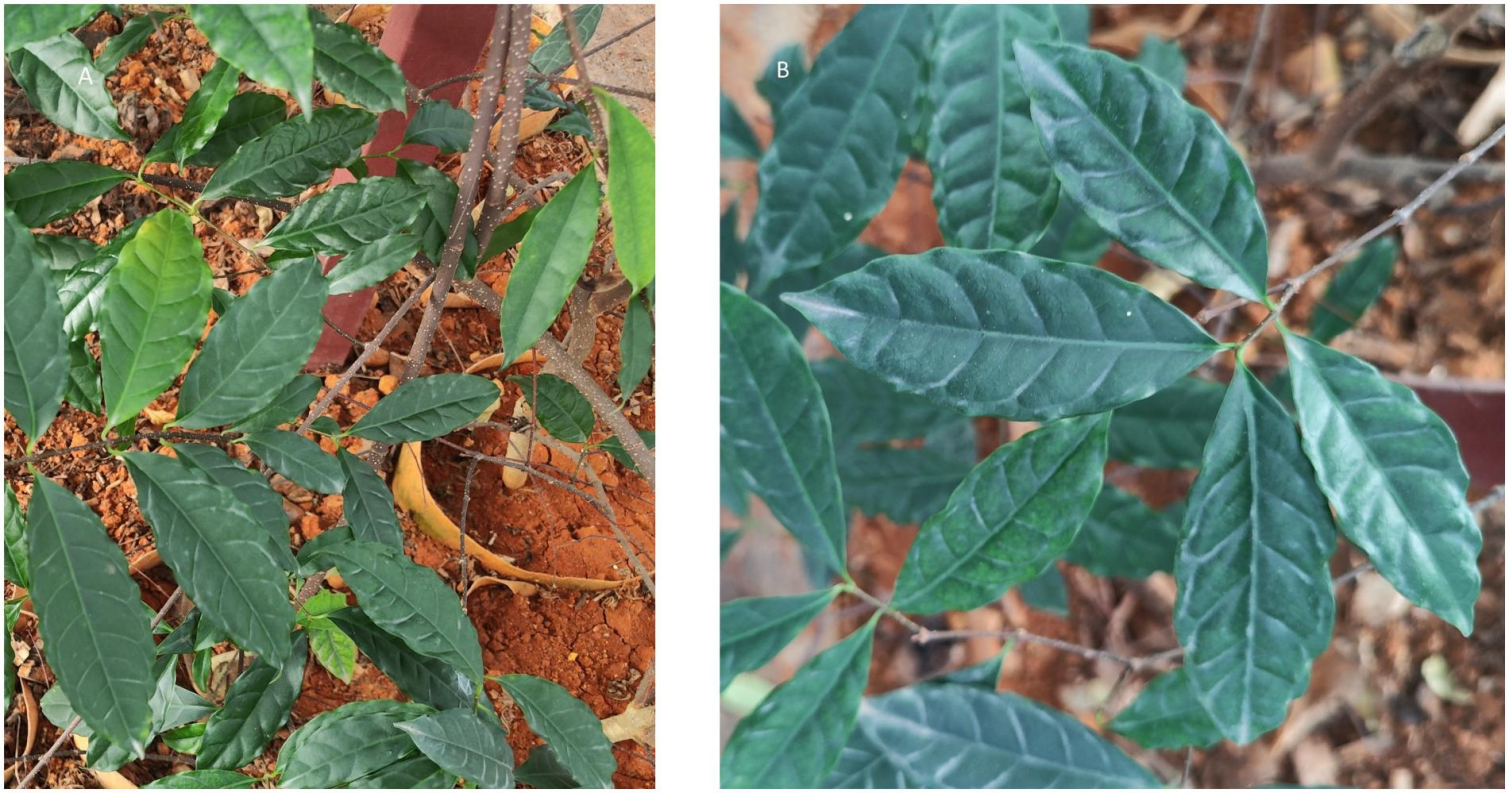
Photograph of *Strophanthus divaricatus* taken at the collection site in Tongmu Town, Jinxiu Yao Autonomous County, Laibin City, Guangxi, China (24.17739°N, 110.008404°E). (A) Branch morphology, showing glabrous, reddish-brown branches with densely distributed grayish-white lenticels. (B) Leaf morphology, showing elliptic or slightly obovate leaf blades (3–10 × 1.5–5 cm) with 4–9 pairs of lateral veins, petioles 5–10 mm long, and adaxial surface dark green and glabrous. All photographs were taken by Ziyao Chen and used with permission.

Mature, pest-free leaves of *S. divaricatus* were collected from Tongmu Town, Jinxiu Yao Autonomous County, Laibin City, Guangxi Zhuang Autonomous Region, China (24.17739°N, 110.008404°E) (Figure 1). Sample collection was conducted with permission from Dr. Xinmin Pan, Guangxi Hongyao Biotechnology Co., Ltd. (email: 40971972@qq.com). A corresponding voucher specimen (accession number: JXHC081) has been deposited in the Herbarium of the Institute of Medicinal Plant Development (IMPLAD), Chinese Academy of Medical Sciences (CAMS), Beijing, China for long-term preservation and future reference (Contact: Guoan Shen; email: agshen@implad.ac.cn).

### DNA extraction and sequencing

Total genomic DNA was extracted using the DNeasy Plant Mini Kit (Cat. 69104, Qiagen) according to the manufacturer’s protocol. Subsequently, high-integrity DNA was fragmented to an average insert size of approximately 300 bp for the creation of the sequencing library. Paired-end sequencing (2 × 150 bp) was performed on the Illumina HiSeq 2500 platform. Raw sequencing data were processed with fastp (v.0.2) to remove adapters and low-quality reads.

### Genome assembly and annotation

High-quality sequences were assembled de novo into the chloroplast genome utilizing the GetOrganelle toolkit (v.1.7.7.0) (Jin et al. 2020). The precision and authenticity of the constructed plastome were confirmed through graphical representation using Bandage v.0.8.1 (Wick et al. 2015). Gene annotation was conducted automatically utilizing GeSeq (Tillich et al. 2017) and CPGAVAS2 (Shi et al. 2019). The completed chloroplast genome map and the maps of cis/trans-splicing genes in *S. divaricatus* were illustrated using CPGView (Liu et al. 2023).

### Phylogenetic analysis

The chloroplast genomes of 22 species within the Gentianales order were obtained from the NCBI GenBank database. Three species of *Psychotria* (Rubiaceae) were designated as outgroup taxa. All chloroplast genome sequences, including the annotated genome of *S. divaricatus*, were imported into PhyloSuite for simultaneous processing and alignment. Multiple sequence alignment was conducted with MAFFT under default settings (Katoh and Standley 2013). The optimal nucleotide substitution model was determined *via* ModelFinder based on the Bayesian Information Criterion (BIC) (Kalyaanamoorthy et al. 2017). Maximum likelihood (ML) phylogenetic reconstruction was performed employing IQ-TREE (Nguyen et al. 2015), utilizing either the standalone version or the web server (Trifinopoulos et al. 2016), with the chosen substitution model (TVM+F + I + G4). Branch support was evaluated with 1,000 ultrafast bootstrap replicates (UFBoot) (Hoang et al. 2018) and 1000 SH-aLRT tests (Felsenstein 1985). The resultant phylogenetic tree was depicted through the iTOL (Interactive Tree of Life) web platform (Letunic and Bork 2024).

## Results

**Figure 2.**
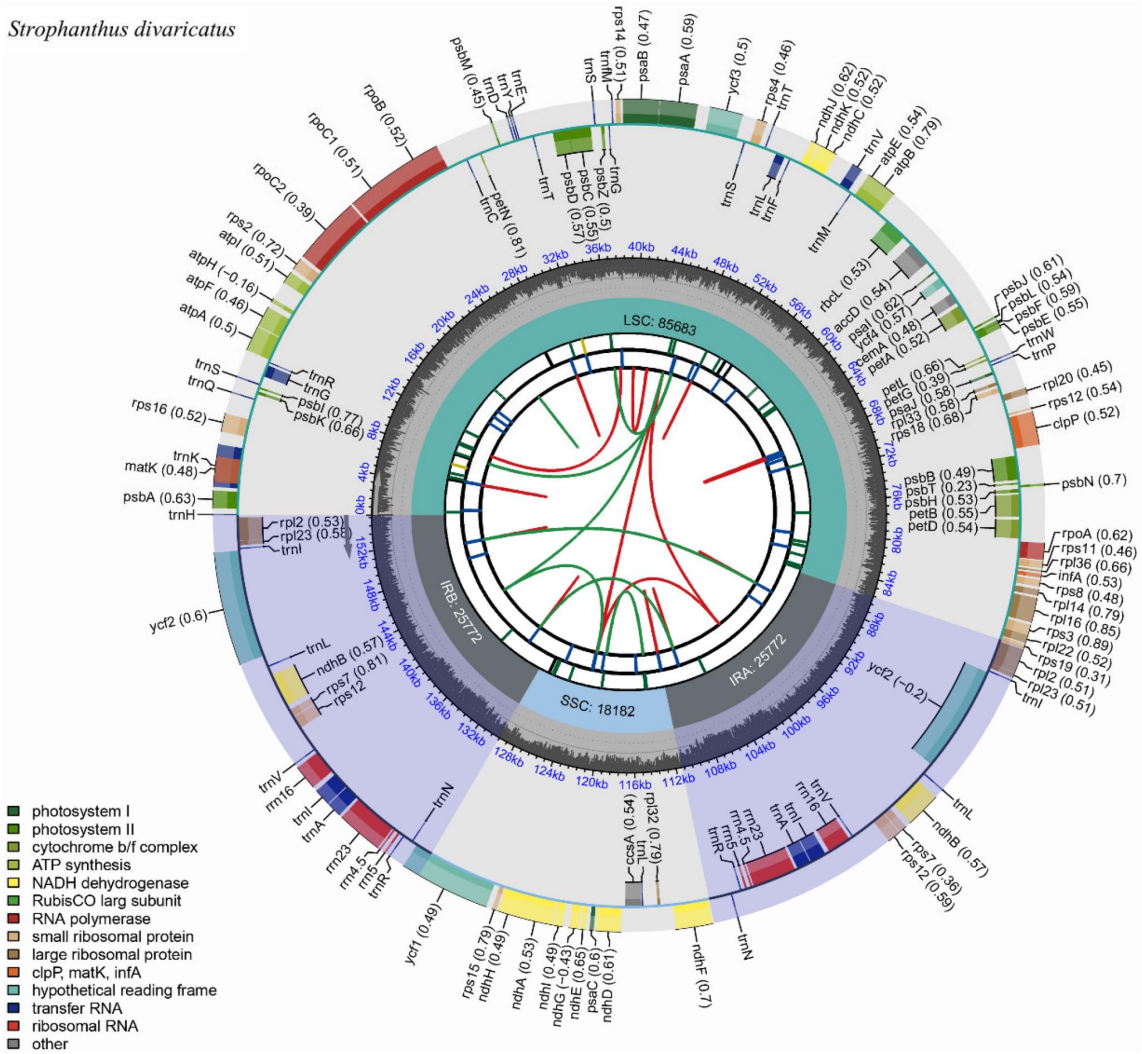
Circular representation of the *Strophanthus divaricatus* chloroplast genome visualized using CPGview. The circular chloroplast genome map comprises concentric tracks depicting key features. From innermost to outermost, these include dispersed repeats (direct in red, palindromic in green); tandem repeats with long repeats shown as blue bars and microsatellites color-coded by repeat unit length (complex: black; mono- to hexa-nucleotide: green to red); the quadripartite structure—LSC (green), SSC (blue), and inverted repeats IRa/IRb (gray); GC content (dark gray) with a 50% threshold; base composition per site between GC and structural tracks; and annotated genes (protein-coding, tRNA, rRNA) color-coded by function. Gene transcription direction corresponds to their position on inner (clockwise) or outer (counterclockwise) circles. Codon usage bias is optionally displayed alongside gene names. A detailed legend is provided in the figure or on the CPGview website.

A total of 13.65 GB of raw sequencing data were generated for *S. divaricatus*. The assembled chloroplast genome was found to be 155,409 base pairs long. The sequencing depth throughout the genome exhibited considerable variation, ranging from 1,358× to 28,967×, with an average depth of 5,131.41× (Figure S1). The chloroplast genome possessed a conventional quadripartite structure consisting of a large single-copy (LSC) region (85,683 bp), a short single-copy (SSC) region (18,182 bp), and two inverted repeats (IRs) of 25,772 bp each. The average GC content was 38.28%. Essential genomic features, including the circular genome map (Figure 2), cis-splicing genes (Figure S2), and trans-splicing genes (Figure S3), were visualized using the CPGView platform.

A total of 130 functional genes were found, comprising 85 protein-coding genes (PCGs), 37 transfer RNAs (tRNAs), and 8 ribosomal RNA genes (rRNAs). In the intergenic areas, there are duplications of six protein-coding genes (*rpl2*, *rpl23*, *rps7*, *rps12*, *ycf2*, and *ndhB*), seven transfer RNAs (*trnN-GUU*, *trnR-ACG*, *trnA-UGC*, *trnI-GAU*, *trnV-GAC*, *trnL-CAA*, and *trnI-CAU*), and four ribosomal RNAs (*rrn5*, *rrn4.5*, *rrn23*, and *rrn16*). The chloroplast genome comprises 23 genes that include introns. Nineteen genes include a solitary intron, comprising eleven protein-coding genes (*rps16*, *atpF*, *rpoC1*, *petB*, *petD*, *rpl16*, *rpl2* (x2)*, ndhB* (x2), and *ndhA*) and eight tRNA genes (*trnK-UUU*, *trnG-UCC*, *trnL-UAA*, *trnV-UAC*, *trnI-GAU* (x2), *trnA-UGC* (x2)). Moreover, two genes (ycf3, clpP) possess two introns. Furthermore, 13 cis-splicing genes and one trans-splicing gene (*rps12*) were found (Figures S2 and S3). The *rps12* gene undergoes trans-splicing, leading to its segmentation into three distinct exons, two of which are replicated within the inverted repeat regions (Figure S3).

**Figure 3.**
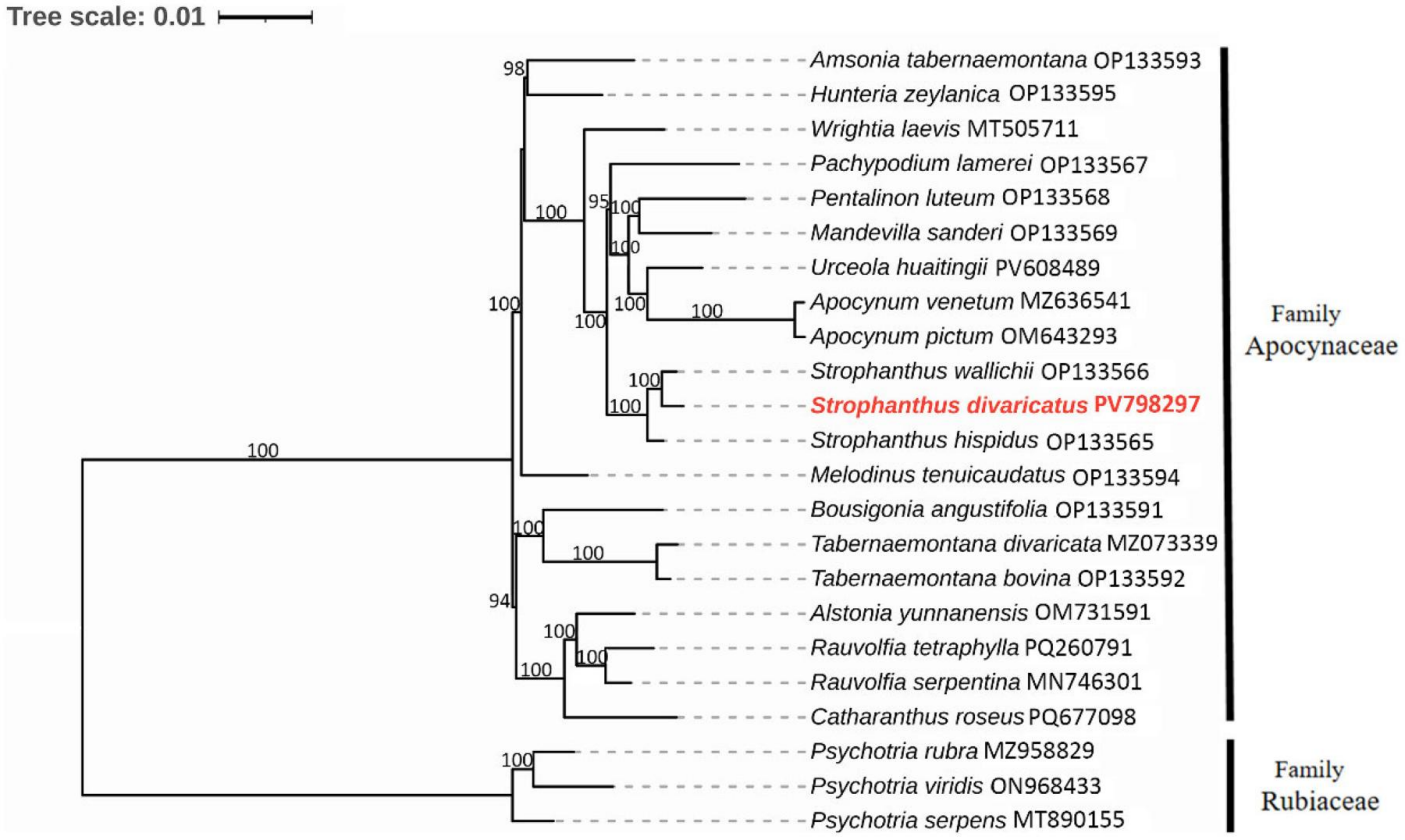
A maximum-likelihood (ML) phylogenetic tree was constructed based on the complete chloroplast genome sequences of *Strophanthus divaricatus* and 22 other species within the order gentianales. Additional sequences used in the analysis include *Amsonia tabernaemontana* (OP133593) (Wang et al. 2023), *Hunteria zeylanica* (OP133595) (Wang et al. 2023), *Wrightia laevis* (MT505711) (Li, Fu, Song 2020), *Pachypodium lamerei* (OP133567) (Wang et al. 2023), *Pentalinon luteum* (OP133568) (Wang et al. 2023), *Mandevilla sanderi (*OP133569) (Wang et al. 2023), *Urceola huaitingii* (PV608489), *Apocynum venetum* (MZ636541), *Apocynum pictum* (OM643293) (Zheng et al. 2022), *Strophanthus wallichii* (OP133566) (Wang et al. 2023), *Strophanthus divaricatus* (PV798297), *Strophanthus hispidus* (OP133565) (Wang et al. 2023), *Melodinus tenuicaudatus* (OP133594) (Wang et al. 2023), *Bousigonia angustifolia* (OP133591) (Wang et al. 2023), *Tabernaemontana divaricata* (MZ073339) (Zhang, Liu, Shan 2021), *Tabernaemontana bovina* (OP133592) (Wang et al. 2023), *Alstonia yunnanensis* (OM731591), *Rauvolfia tetraphylla* (PQ260791), *Rauvolfia serpentina* (MN746301), *Catharanthus roseus* (PQ677098), *Psychotria rubra (*MZ958829) (Geng et al. 2022), *Psychotria viridis* (ON968433) (Varani et al. 2022), and *Psychotria serpens* (MT890155).

A Maximum Likelihood (ML) phylogenetic analysis utilizing protein-coding genes from the chloroplast genomes of 23 species in the order Gentianales was conducted to investigate the evolutionary position of *S. divaricatus* within the family Apocynaceae. This study showed that three *Strophanthus* species were grouped into a strongly supported monophyletic clade, with *S. divaricatus* and *S. wallichii* identified as sister taxa in the phylogenetic analysis.

## Discussion and conclusion

In this study, we successfully assembled the complete chloroplast genome of *S. divaricatus*. The *S. divaricatus* chloroplast genome exhibits structural and gene content features similar to those of *S. wallichii* and *S. hispidus,* including the presence of non-canonical start codons in the *rps19* and *ndhD* genes (Wang et al. 2023). Such nonstandard initiation codons are relatively common in plant plastomes and may be associated with gene expression regulation, adaptive significance, and phylogenetic patterns (Chi et al. 2018). The three *Strophanthus* species formed a monophyletic clade, with *S. divaricatus* showing a closer genetic relationship to *S. wallichii*. In conclusion, this study substantially enhances the chloroplast genomic resources of *S. divaricatus*, addressing a critical gap in the genetic information of this species. It provides a solid foundation for investigating the genetic diversity and phylogenetic relationships within the genus *Strophanthus* and the family Apocynaceae. The newly obtained chloroplast genome sequence offers valuable support for future species identification, population genetics research, and conservation efforts.

## Supplementary Material

Supplemental Material

## Data Availability

The complete chloroplast genome sequence of *Strophanthus divaricatus* generated in this study has been deposited in the NCBI database under the accession number PV798297.1. The associated BioProject, BioSample, and Sequence Read Archive (SRA) numbers are PRJNA1290534, SAMN49923244, and SRR34532193, respectively. All data are publicly available and can be accessed *via* the NCBI database (https://www.ncbi.nlm.nih.gov/).
