# Supplementary material for: The complete chloroplast genome sequence of the medicinal plant Strophanthus divaricatus (Lour.) Hook. & Arn. (1837) and its phylogenetic analysis
Source: Mitochondrial DNA B Resour. 2026 Mar 5;11(4):510–4. doi: 10.1080/23802359.2026.2638668 (PMC12973815; doi:10.1080/23802359.2026.2638668)

**Figure S1.** Read coverage across the chloroplast genome of *Strophanthus divaricatus*. The horizontal axis denotes genomic positions, whereas the vertical axis indicates the corresponding sequencing depth.

**Figure S2**. Cis-splicing genes identified in the chloroplast genome of *Strophanthus divaricatus*. The orientation of each gene is indicated by a directional arrow. Genes are represented by black segments for exons and white segments for introns. Gene names are displayed to the left of the corresponding arrows, and genomic coordinates are shown below each gene representation.

**Figure S3**. Diagrammatic representation of the trans-splicing gene *rps12* in the chloroplast genome of *Strophanthus divaricatus*.

## Figure S1


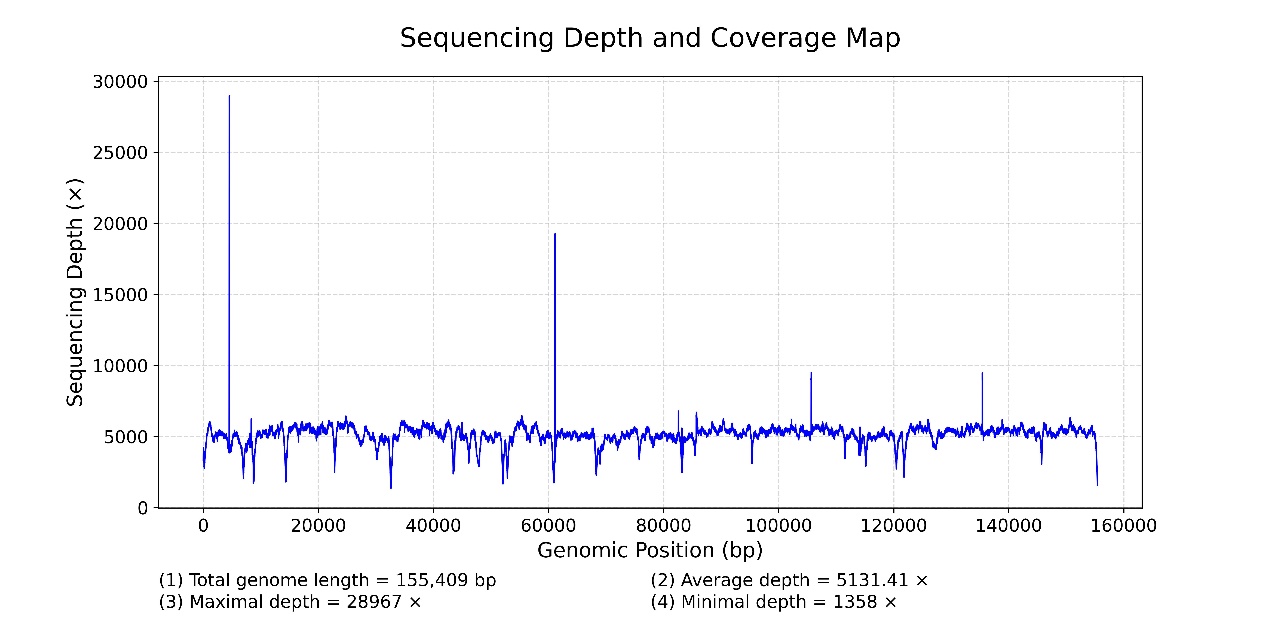


## Figure S2


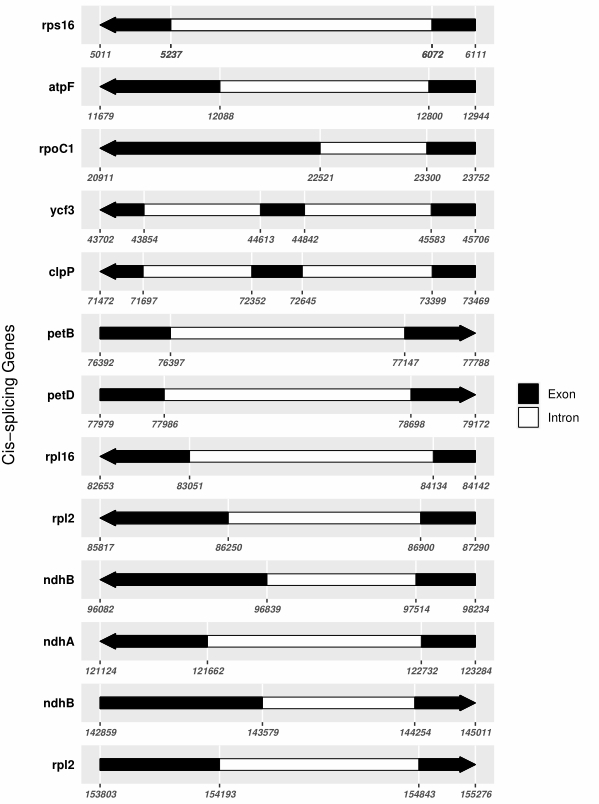


## Figure S3


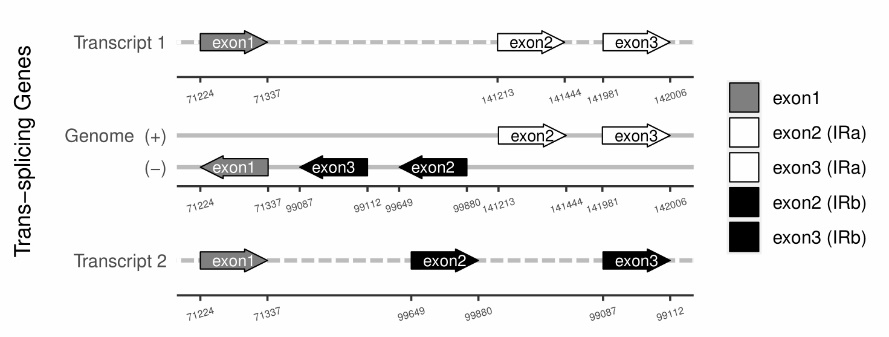

Supplement: Supplemental Material [file TMDN_A_2638668_SM7723.docx]
